# Supplementary material for: Brazilian female researchers do not publish less despite an academic structure that deepens sex gap
Source: PLoS One. 2022 Aug 29;17(8):e0273291. doi: 10.1371/journal.pone.0273291 (PMC9423670; doi:10.1371/journal.pone.0273291)
Supplement: S1 Fig — Impact metrics: number of citations, RCR, FCR and Altmetric, considering both male and female researchers occupying first and last positions on papers. Absolute and Relative values are demonstrated side by side. (PDF) [file pone.0273291.s001.pdf]

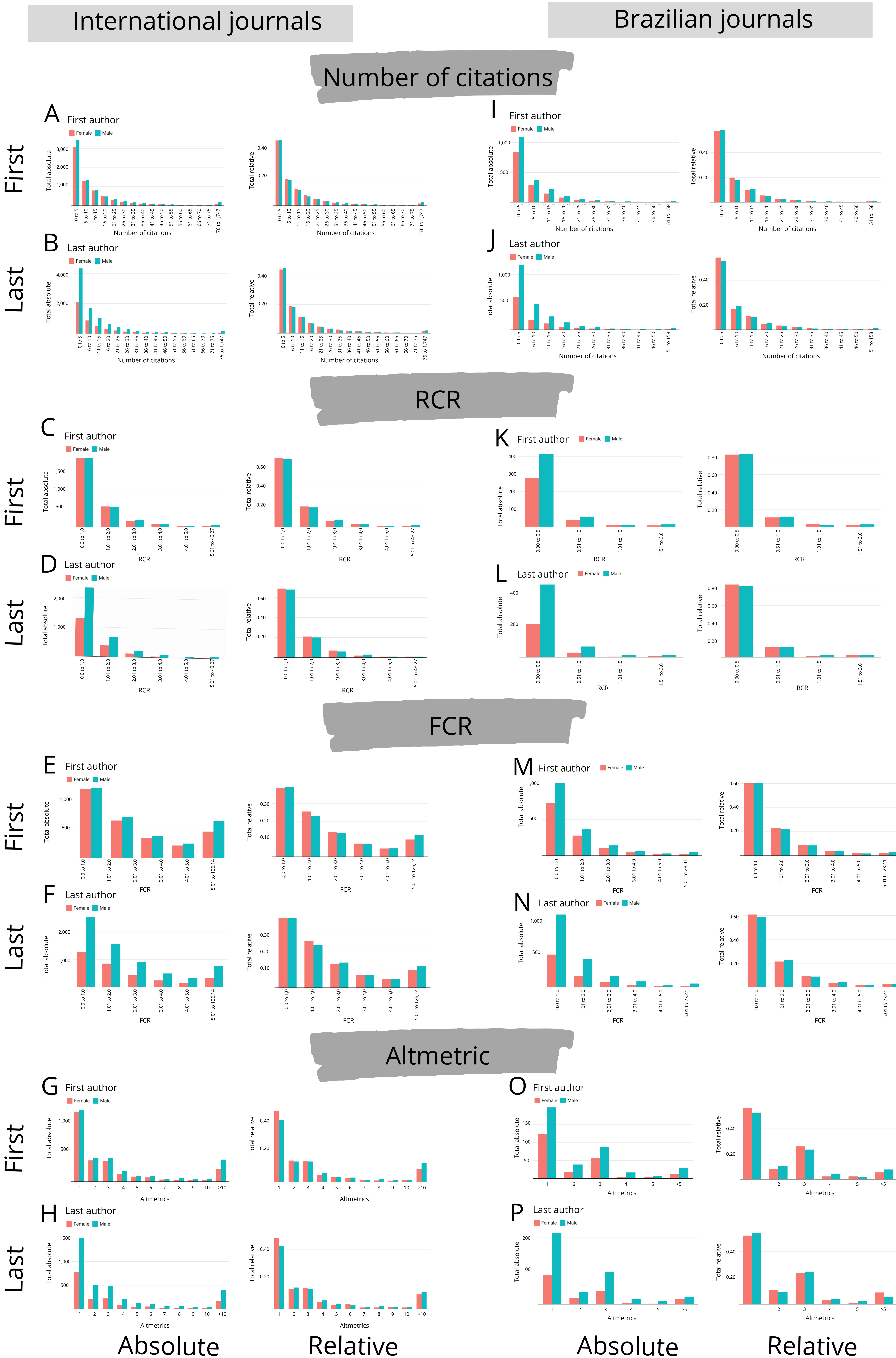

International journals
Brazilian journals
Number of citations
A First author
B Last author
I First author
J Last author
Female
Male
Total absolute
Total relative
Number of citations
RCR
C First author
D Last author
K First author
L Last author
FCR
E First author
F Last author
M First author
N Last author
Altmetric
G First author
H Last author
O First author
P Last author
Altmetrics
First
Last
Absolute
Relative
