# Supplementary material for: Brazilian female researchers do not publish less despite an academic structure that deepens sex gap
Source: PLoS One. 2022 Aug 29;17(8):e0273291. doi: 10.1371/journal.pone.0273291 (PMC9423670; doi:10.1371/journal.pone.0273291)
Supplement: S2 Fig — Fig 1. Male to the female ratio by year with absolute data considering one as an equal ratio value. Values higher than one evidence more male researchers and lower evidence more female researchers as first or last authors. Fig 2. Male to the female ratio by year with relative data considering one as an equal ratio value. Values higher than one evidence more male researchers and lower evidence more female researchers as first or last authors. (DOCX) [file pone.0273291.s002.docx]

**The gender gap in Brazilian Entomology: an analysis of the academic scenario**

Male to female ratios by year with absolute (Fig 1) and relative (Fig 2) values. Articles were searched on September 1st 2021, in the Dimensions Website (https://app.dimensions.ai/exports) and all articles published were explored on Brazilian Entomology Journals.

We analyzed both absolute and relative number of female and male names for comparisons of the number of papers and citations. We calculated the relative numbers by dividing the value of each sex for a certain year (for number of papers) or value of the impact metric (for citations) by the total records of that sex, for example, relative(women) = women(year)/women(all years).


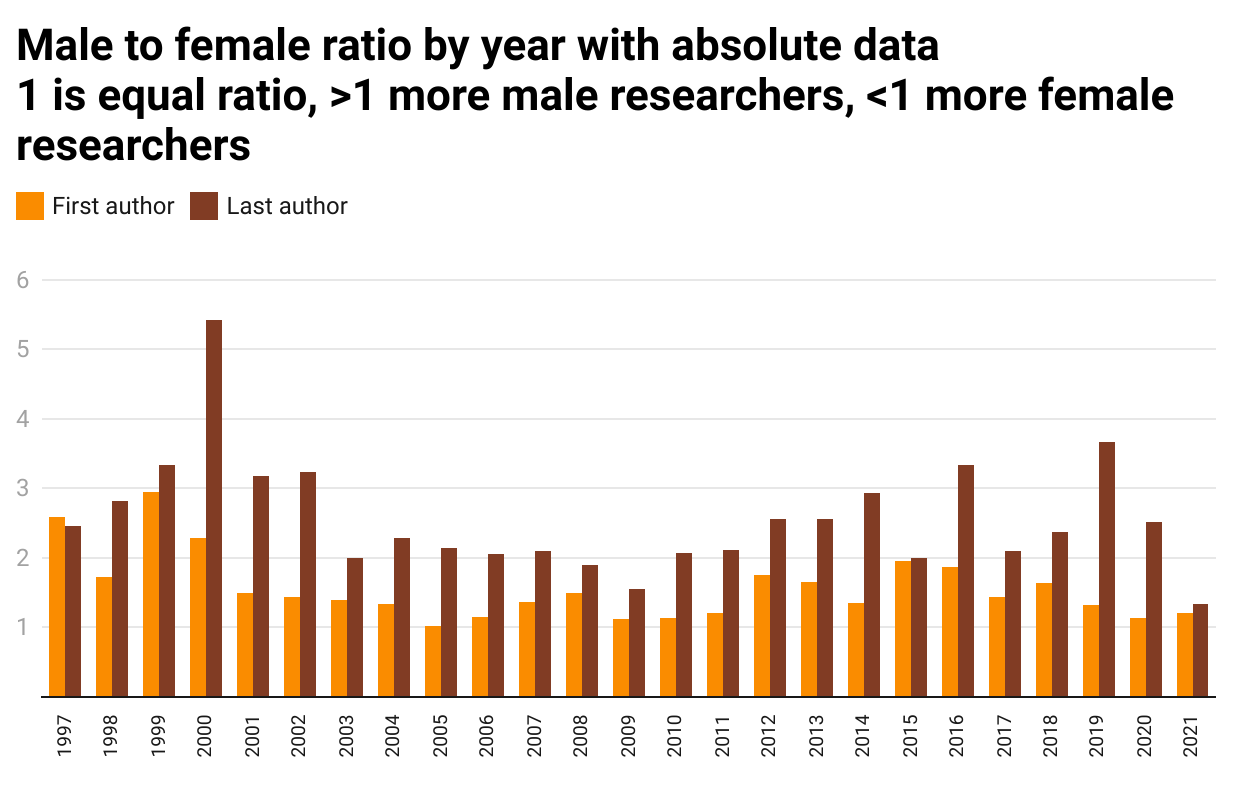


Fig 01. Male to the female ratio by year with absolute data considering one as an equal ratio value. Values higher than one evidence more male researchers and lower evidence more female researchers as first or last authors.


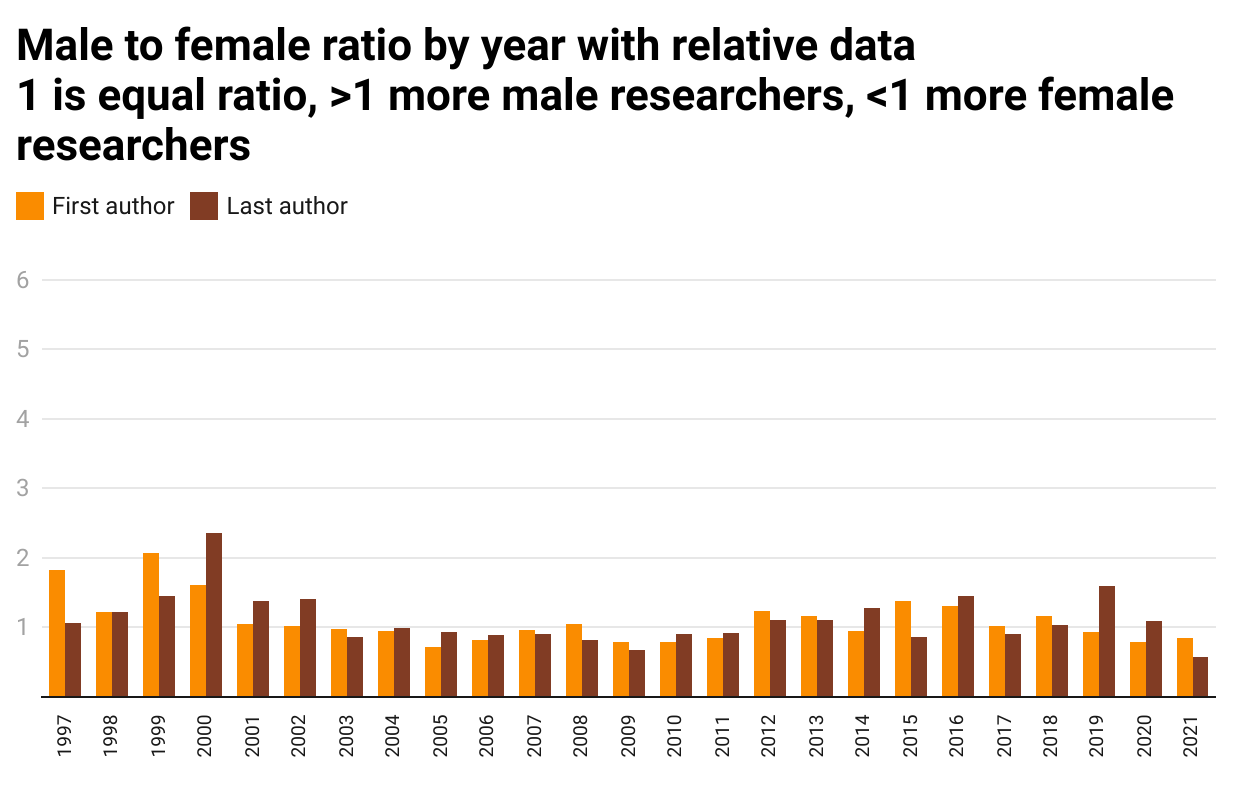


Fig. 02. Male to the female ratio by year with relative data considering one as an equal ratio value. Values higher than one evidence more male researchers and lower evidence more female researchers as first or last authors.
