# Supplementary material for: Brazilian female researchers do not publish less despite an academic structure that deepens sex gap
Source: PLoS One. 2022 Aug 29;17(8):e0273291. doi: 10.1371/journal.pone.0273291 (PMC9423670; doi:10.1371/journal.pone.0273291)
Supplement: S1 File — (DOCX) [file pone.0273291.s005.docx]

Data Availability Statement - **Brazilian female researchers do not publish less despite an academic structure that deepens sex gap**

The datasets analyzed during the current study are available from the Capes portal at https://geocapes.capes.gov.br/geocapes/ and https://dadosabertos.capes.gov.br/dataset?organization=diretoria-de-avaliacao, and from Dimensions at <https://www.dimensions.ai>. At this last (Dimensions) some restrictions may apply considering terms and conditions. Data are however available from the authors upon reasonable request and permission of Dimensions (approved proposed project (DIM-024)) and can also be accessed through a personal or institutional registration at Dimensions portal. Thus, none of the used third party data had any special access privileges that others would not have. All data is available on the websites that we provided and anyone can have free access with no need to further details as the websites are enough.
