# Supplementary material for: Brazilian female researchers do not publish less despite an academic structure that deepens sex gap
Source: PLoS One. 2022 Aug 29;17(8):e0273291. doi: 10.1371/journal.pone.0273291 (PMC9423670; doi:10.1371/journal.pone.0273291)
Supplement: Attachment — Submitted filename: Comments Paper Female_PLOS ONE.pdf [file pone.0273291.s007.pdf]

Paper

Female researchers do not publish less despite an academic structure that deepens sex gap

Comments

Interesting and current topic applied to a specific area of investigation. It can inspire other researchers from other areas to carry out similar studies to verify if the conclusions obtained are similar in other areas of knowledge.

Very careful description of Materials and Methods.

Extensive list of bibliographic references and recent publications.

Very good results discussion.

Minor fixes:

1. Check the entire text “Brazilian Academy of Science” or ”Sciences”.

2. Any explanation for the peak of publications to be between the years 2003 to 2012 and remain more stable from there?

3. Table 2 - absolute values sum 30 and not 31 as indicated in the text.

4. Conclusions are missing. some sentences from the discussion should be included in a new “Conclusions” section.
