# Supplementary material for: Brazilian female researchers do not publish less despite an academic structure that deepens sex gap
Source: PLoS One. 2022 Aug 29;17(8):e0273291. doi: 10.1371/journal.pone.0273291 (PMC9423670; doi:10.1371/journal.pone.0273291)
Supplement: Attachment — Submitted filename: Response letter.docx [file pone.0273291.s008.docx]

Dear Editor,

We want to thank you and the two anonymous reviewers for the valuable and constructive

feedback on the submitted manuscript. We have addressed most of reviewer’s comments. Some of them we did not, yet we justified below. Please see our detailed responses (in blue) to each comment (in black). In addition to addressing the suggestions by the reviewers.

We hope that the revised manuscript fulfils the expectations for publication in PlosOne.

Best regards,

Juliana Hipólito and colleagues

Comments from Reviewer 01:

Interesting and current topic applied to a specific area of investigation. It can inspire other

researchers from other areas to carry out similar studies to verify if the conclusions obtained are

similar in other areas of knowledge.

Very careful description of Materials and Methods.

Extensive list of bibliographic references and recent publications.

Very good results discussion.

RESPONSE: Thank you for your valuable and constructive feedback. We are very glad about all positive comments.

Minor fixes:

1. Check the entire text “Brazilian Academy of Science” or ”Sciences”. Done

2. Any explanation for the peak of publications to be between the years 2003 to 2012 and remain more stable from there?

For any scientific field there is an increase of publications, as we showed for example in Fig 5 (for all journals), which makes this question pertinent because it does not follow this general trend. The comment refers to Fig 3, related to the Brazilian journals. The increase in 2003 is partly related to the general trend, also partly because the Revista Brasileira de Entomologia appears in the dataset in 2002 - another "jump" happens in 2008 with the addition of data from EntomoBrasilis in the database. The number of articles indeed lessened by 2012, but it does not relate to removal of journals in the dataset. The most relevant journals, Revista Brasileira de Entomologia and Neotropical Entomology, both decrease their number of papers by 2012. We are not completely sure for what generate those patterns yet, seems to have some explanations that are beyond our objectives, although super interesting to be analyzed in future studies.

3. Table 2 - absolute values sum 30 and not 31 as indicated in the text. Corrected

4. Conclusions are missing. some sentences from the discussion should be included in a new

“Conclusions” section. We included a conclusions section as suggested. Thank you very much.

Comments from Reviewer 02:

Please consider explaining why women are referred to as a minority, since they represent the majority of the world's population.

RESPONSE: We included more explanation on that on the first paragraph.

A trendline of the average could be interesting

RESPONSE: We appreciate reviewer comment and we made the legend clear. However we do not consider that we can add a trendline of average as in each year we have the total number of researchers instead of a population data in each year.

A plot that describes the relation between researchers in ABC and researchers in Entomology would be interesting

Response: We appreciate reviewer comments, yet we already made this comparison in a previous paper [1] where we included this relation between researchers in different academic stages. As this was the focus of our previous paper, and at this present paper, this could be incomplete, we would instead not include a plot that described this relation here.

1.	Hipólito J, Shirai LT, Halinski R, Guidolin AS, da Silva Dias Pini N, Soares Pires CS, et al. The Gender Gap in Brazilian Entomology: an Analysis of the Academic Scenario. Neotrop Entomol. 2021 [cited 12 Nov 2021]. doi:10.1007/s13744-021-00918-7

It would be interesting to add a plot of first author/last author, or similar, to establish a relation

RESPONSE: We really appreciate reviewer suggestion. We included this plot as a supplementary file

Figure 5. Please standardize. Please clarify if relative(women)=women/(women+men) or relative(women)=women(year)/women(all years)

RESPONSE: Absolutely, this is very relevant. As the same calculation was used for all relative values we report on the paper, we added the equation to the Material and Methods section, where it was already written but perhaps not so clearly. If the editor and/or reviewer think(s) it is relevant to add to the figure legend, we add during proofs to both Figure 3 and 5
