# Supplementary material for: Brazilian female researchers do not publish less despite an academic structure that deepens sex gap
Source: PLoS One. 2022 Aug 29;17(8):e0273291. doi: 10.1371/journal.pone.0273291 (PMC9423670; doi:10.1371/journal.pone.0273291)
Supplement: Attachment — Submitted filename: response letter_2version.docx [file pone.0273291.s009.docx]

Dear Editor, we really appreciate reviewers comments although a few we do not agree. Below we comment

Review Comments to the Author

Please use the space provided to explain your answers to the questions above. You may also include additional comments for the author, including concerns about dual publication, research ethics, or publication ethics. (Please upload your review as an attachment if it exceeds 20,000 characters)

Reviewer #1: The authors made the necessary changes to the paper and it is now suitable for publication. As a final remark, the reviewer suggests using gender instead of sex in the title, since the social term "gender" is more suitable than the biological "sex", to the reviewer's best knowledge.

Answer: We appreciate reviewer comments, but we, unfortunately, did not access gender in our study as gender is socially constructed and is not binary. As we just identified names instead of a person’s response, we do not see as the most appropriate term the use of term “gender.”

Reviewer #3: The paper “Female researchers do not publish less despite an academic structure that deepens sex gap” describes gender inequality in Brazilian science in terms of the presentation of women in key / authoritative positions despite the fact of equal productivity from a bibliometrics viewpoint. The results are based on a solid ground formed from three sources: (a) the structure of the Brazilian Academy of Science; (b) the composition of editorial boards of the Brazilian journals; and (c) the positions of males/females in the bylines of papers published by Brazilian researchers. The sample is limited by Entomology subject area; bibliometric analyses were carried out using the Dimensions database. Applied approaches are thoroughly detailed, as well as the samples used.

The paper is believed to contribute to our knowledge on gender distribution and productivity not only because it concerns poorly studied region and one more subject area, but also due to substantive discussion of what should be made to increase inclusivity, diversity, and equality. The authors proposed several steps including raise of awareness of the problem, introducing some normalized values considering motherhood, the age of children, other family responsibilities, etc. to increase reasonable recognition of male/female contribution in science. I think the revised version of the manuscript is worth publishing after addressing several minor issues.

Answer: We really appreciate reviewer comments

Comments.

1. Title. Now the title lacks both mentioning entomology subject area and Brazilian science. I think they should be included in the title to make it more precise.

Answer: We included Brazilian at the title and entomology at the keywords. As the title is already a bit larger we’d rather not to include entomology at the title.

2. **Pp. 14-15, Brazilian journals description. It will be useful to add some more information on Brazilian entomological journals, like their ISSNs, websites, indexing systems, the language of the articles, etc., to make readers’ perceptions more informed.**

Answer: We added this information on a supplementary table. Supplementary material 3.

3. **As I understand, some Brazilian journals may be international ones, include international members, and thus may represent not only Brazilian, but also international models of gender distribution in entomology. Thus, a more detailed explanation of the compound of editorial boards in Brazilian journals is needed.**

Answer: We added more information on this point at the material and methods. We agree with reviewer point of view.

4. **The other interesting point is to compare male/female distribution in Brazilian journals and, e.g., top-10 international entomological journals. For instance, in 10.3103/S0147688218040123 it was found that women were underrepresented in a set of analyzed national journals on Earth Sciences while international ones followed other models. Is there Brazilian specific in this aspect?**

Answer: We added another supplementary material with the top ten international journals form SciMago (supplementary material 4) and expand this discussion on the discussion section.

3. P. 22: “if we, as a community, do not admit that hegemony of any kind does not make better science.” – However, sometimes the causes of inequality are reasonable and should not be solved by a simple establishment of proportions. See, e.g., 10.1016/S0140-6736(03)12528-7.

Answer: I agree with reviewer comment and really appreciate suggestion. I added a note about it: “if we, as a community, do not admit that hegemony of any kind does not make better science. Still one may point that the causes of inequality are reasonable and should not be completely solved by a simple establishment of proportions [59].”

4. P. 9: “Embrapa Arroz e Feijão, Santo Antônio de Goiás, GO, Brazil.” – remove dot character in the end of the line.

Answer: Excluded

5. P. 10: “degree stages in STEM” – please, explain the abbreviation on first mention.

Answer: We included the explanation

6. P. 14: The reference to the Dimensions Website is indicated twice in this page. I believe the second mention can be removed.

Answer: We excluded the second as suggested

Reviewer #4: The topic is interesting and the new version is understandable and easy to read. All the changes requested by the reviewers were made.

Answer: We appreciate reviewer comments
